# Controlled adsorption of polyurethane onto chlorine-modified carbon nanotubes for enhanced mechanical and electrical properties of nanocomposites

**DOI:** 10.1039/d5na00653h

**Published:** 2025-10-20

**Authors:** Yadienka Martinez-Rubi, Hao Li, Kiran Mungroo, Michael B. Jakubinek, Behnam Ashrafi, Zygmunt J. Jakubek, Liliana Gaburici, Christopher Kingston

**Affiliations:** a Quantum and Nanotechnologies Research Centre, National Research Council Canada Ottawa ON Canada Yadienka.Martinez-Rubi@nrc-cnrc.gc.ca; b Aerospace Research Centre, National Research Council Canada Montreal QC Canada; c Metrology Research Centre, National Research Council Canada Ottawa ON Canada

## Abstract

Maximizing the performance of carbon nanotubes (CNTs) in polymer nanocomposites remains a significant challenge, and fabrication methods often fall short of achieving the desired properties. From an application perspective, enhancing both mechanical properties and electrical conductivity is particularly desirable, yet simultaneously improving multiple properties is often difficult – the high loadings in dispersed CNT composites needed for better conductivity tend to impede processing and mechanical properties. Herein, a one-step filtration method with solubility modulation to control the adsorption of thermoplastic polyurethane (TPU) onto a commercial-grade single-wall CNT (SWCNT) powder is employed. The impact of solvent choice on interfacial interactions, the properties and microstructure of SWCNT–TPU nanocomposite sheets is highlighted. The results demonstrate that sonochemically generated chlorine, known to enhance the electrical conductivity of SWCNT assemblies, also improves interfacial interactions and the adsorption capacity of the nanotubes for TPU leading to significant enhancements in electrical conductivity, Young's modulus, and failure strength of the nanocomposites without compromising ultimate strain. Analysis of effective adsorption isotherms gives new insights into the mechanisms driving interfacial interactions and a model detailing various adsorption regions is proposed. Saturation differences in SWCNT adsorption sites, contingent on processing conditions, account for observed trends and determine the optimal SWCNT/TPU ratio and factors affecting macroscopic properties. Furthermore, insights from effective adsorption isotherms can guide the optimization of the fabrication method to enhance properties.

## Abstract 2

Controlled adsorption optimizes nanocomposite sheet properties by enhancing interfacial interactions. Effective adsorption isotherms help understand trends, TPU–SWCNT interactions, and factors affecting mechanical properties and conductivity.

## Graphical abstract

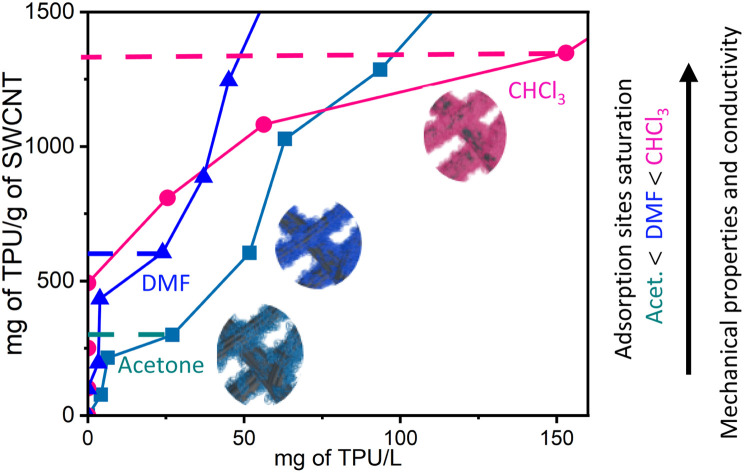

## 1. Introduction

Carbon nanotubes (CNTs) possess multiple compelling properties, such as low density, high strength, high electrical and thermal conductivity that make them attractive as reinforcement materials for multifunctional composites.^1^ Notable features for consideration as reinforcements are their high aspect ratio (length to diameter ratio), which give them bendability, and their high specific surface area, which provides for ample contact area with a matrix to enhance interfacial effects. It has been long recognized that fabricating CNT-polymer composites with optimal mechanical properties and conductivity involves several key factors. This includes the selection of the type of CNT and polymer matrix, the CNT dispersion and distribution within the composite, the use of surface modification to improve compatibility, the CNT loading, aspect ratio and orientation.^2^,^3^ Significant efforts can be found in the literature towards the development of homogeneous nanocomposites with well dispersed nanotubes, usually limited to low nanotube contents (less than 5–10 wt%).^4–6^ When using melt blending, for example, this limit in CNT content arises from a significant increase in viscosity with increasing CNT content,^7^ whereas in solution-based methods, higher nanotube loadings increase the difficulty of controlling nanoparticle agglomeration during solvent removal. Moreover, architectures with homogeneously distributed CNTs don't always lead to an effective enhancement in the mechanical and physical properties, while the fabrication of nanocarbon-reinforced composites with anisotropic distributions such as laminates, alignment, and stochastic networks, has been reported to break through such limitations and improve the overall performance of composites.^8^ In this context, polymer nanocomposites in the form of fabriclike sheets that incorporate a high content of nanotubes have attracted increasing attention.^9^ In nanocomposite sheets, 1-dimensional (1D) CNTs form a 2D interconnected network, with random distribution or aligned along a single orientation, rendering anisotropic properties. These nanocomposites are commonly fabricated by a two-step method, which involves CNT preform fabrication followed by polymer infiltration. Other fabrication methods include solution casting, layer-by-layer deposition, and *in situ* polymerization. The topic has been recently reviewed and the advantages and disadvantages discussed.^10^ For example, *in situ* polymerization is limited to certain types of polymers, and the thickness and filler fraction are difficult to control through vacuum assisted polymer infiltration.

Solubility modulation is a simple technique where, upon interaction with a nonsolvent, polymer chains in solution are driven to deposit onto the nanocarbon surface and multifunctional nanocomposites with high content of nanocarbons can be recovered.^11,12^ Several examples are found in the literature were nonsolvent-induced phase separation (NIPS), has also been reported for the fabrication of polymer nanocomposites. These include, superhydrophobic PVC porous composite films,^13^ thermoplastic polyurethane (TPU)-nanocomposites^14,15^ and foams^16^ for electromagnetic interference shielding. Similarly, *via* solubility modulation, we have used a one-step vacuum filtration method to fabricate stretchable SWCNT–TPU nanocomposites with improved mechanical properties, up to 1.8 GPa Young's modulus and 80 MPa tensile strength, for flexible thermoelectric materials.^17^ With this method, TPU-coated nanotubes/bundles are formed in solution and are readily recovered by vacuum filtration to produce nonwoven nanocomposite sheets with high content of entangled SWCNTs and controlled composition. In particular, TPUs, which are characterized by low stiffness and stress at low strain but high ductility and toughness, have been shown to be promising for balancing the mechanical properties of CNT reinforced polymers for the production of stiff, strong, yet tough thermoplastic polymer composites.^18^ Another practical advantage of this on-step filtration method is that the CNT-coated suspension can be readily vacuum-filtered in about (5 to 15) min, even at high TPU concentrations, and the fraction of polymer not associated with the nanotubes and remaining in the solvent mixture can be removed in the filtrate. Moreover, as shown for multi-wall CNTs (MWCNTs), where the MWCNT/TPU weight ratio was precisely controlled, nanocomposites exhibited optimal mechanical properties (strength and modulus) and electrical conductivity within a specific, narrow, composition range.^12^

Similar to our observations for MWCNT–TPU sheets,^12^ a nonmonotonic dependence of mechanical properties on the nanofiller fraction has also been reported in other polymer nanocomposites with high content of nanocarbons.^19^ The current understanding and general principles of interphase formation have been recently highlighted.^20^ The authors argue that a continuous interphase (a co-continuous network of dramatically altered polymer chains) might be an ideal structure for the mechanical reinforcement of nanocomposite materials. Zhao *et al.*, recently developed nanocomposite films with highly ordered layered structures exhibiting ultrahigh mechanical properties.^21^ The strength and modulus of these films increased with filler content up to 25 wt%, before decreasing with further increases in nanofiller content. They concluded that adjusting the nanofiller weight percentage induces the interlayer distance to a critical value, resulting in the formation of a critical interphase. Consequently, the maximum point in the nonmonotonic dependence of mechanical properties on nanofiller content occurs when the ratio of the interphase and the non-interphase material reaches this critical value.

Compared with MWCNT–TPU sheets, SWCNT–TPU sheets fabricated using the same method displayed a different behavior, lacking a well-defined composition that maximized strength and modulus.^17^ Multifunctional nanocomposites are attractive for many current and emerging applications, with a nanotube–polymer sheet format offering advantages for handling and integration.^22^ Therefore, identifying processing conditions that enable the construction of a co-continuous CNT–polymer network with optimal mechanical properties and functionalities – a critical interphase – is essential. In solution-based fabrication methods, the conditions that govern the formation of the surface layers at the interface with the solid reinforcement are crucial in determining the final composite properties. Processing parameters such as sonication and solvent choice influence the degree of CNT debundling and disentanglement, which in turn affect the available surface area for polymer adsorption and coprecipitation. The processing conditions that optimize interphase and nanotube entanglement in the final nanocomposite sheets are expected to yield the best mechanical and conductivity properties.

Here we show the effect of three different TPU solvents: acetone, DMF and CHCl_3_, on the fabrication of high-nanotube-content SWCNT–TPU sheets using the one-step filtration method. We demonstrate that the TPU-solvent employed in the solvent/nonsolvent mixture has a significant impact on the mechanical properties, the trends in these properties with compositions, and on the electrical conductivity. We also show that adsorption studies (deposition of TPU onto the surface of SWCNTs) are a valuable tool to understand the observed differences as well as to demonstrate improved SWCNT–polymer interactions due to surface modification, and thus to optimize the fabrication method.

## 2. Materials and methods

The thermoplastic polyurethane was a methyl diisocyanate (MDI)-based polyester–polyurethane (Pine Brook, NJ) with a density of 1.19 g cm^−3^ and a Shore hardness of 85A. Single-wall carbon nanotubes (SWCNTs) under the brand name Tuball™ (OCSiAl, Luxembourg) were purchased and used as-received. These SWCNTs have diameters of (1 to 3) nm and lengths of (1 to 5) μm.^23^ According to the manufacturer, the SWCNT powder contains less than 15% metal impurities (*i.e.*, >85% carbon purity).

### 2.1. SWCNT–buckypaper (BP) and nonwoven SWCNT–TPU composite sheets

Nonwoven SWCNT–TPU composite sheets with high loadings of SWCNTs were fabricated by adapting the previously reported one-step filtration method.^12,17^ SWCNT–TPU nanocomposite sheets with a wide range of controlled compositions are recovered by vacuum filtration using a suspension of TPU-coated SWCNT that is obtained in a TPU solvent/non-solvent mixture. Different TPU solvents were used (acetone, DMF and CHCl_3_), with methanol as nonsolvent in all cases. Buckypapers (SWCNT–BPs, 100 wt% SWCNTs) were also prepared from the different solvent mixtures. See Table S1in the SI.

### 2.2. Acetone/methanol (acetone-nanocomposites)

30 mg of SWCNTs were dispersed in 50 mL of acetone using an IKA 25 Ultra-Turrax high-speed disperser (at 6000 rpm for 1 min then 7000 rpm for 1 min), followed by 1 hour of bath sonication, followed by 10 min of horn sonication (Misonix, 30% output, 67% duty cycle) and another 30 min of bath sonication. The TPU dissolved in 50 mL of acetone was then combined with the SWCNTs followed by another 10 min of tip sonication and 30 min of bath sonication. The volume of the suspension was then completed to 110 mL with acetone. 70 mL of methanol was then slowly added to the suspension while running the high-speed disperser followed by 30 min of bath sonication. 10 min of horn sonication was then carried out while slowly adding an additional 50 mL of methanol during this process. Lastly, another 30 min of bath sonication was carried out.

### 2.3. DMF/methanol (DMF-nanocomposites)

30 mg of SWCNTs were dispersed in 75 mL of DMF. TPU dissolved in 10 mL of DMF was combined with the SWCNTs and the volume of the suspension was then completed to 150 mL with DMF. This suspension was then combined with a total of 350 mL of methanol. The high-speed disperser, bath and horn sonication steps were applied as described above.

### 2.4. Chloroform/methanol (CHCl_3_-nanocomposites)

30 mg of SWCNTs were dispersed in 50 mL of chloroform. TPU dissolved in 10 mL of chloroform was then combined with the SWCNTs and the volume of the suspension was then completed to 75 mL with chloroform. This suspension was then combined with a total of 150 mL of methanol. The high-speed disperser, bath and horn sonication steps were applied as described above.

The TPU-coated SWCNTs prepared with each of the solvents were recovered as non-woven sheets by vacuum filtration through a PTFE membrane (1.2 μm pore size) using a Venturi air pump. Filtration was completed quickly, within (5 to 15) min. The wet nanocomposite sheets were immediately sandwiched between parchment and filter papers and dried flat at room temperature overnight, after which they were peeled from the filter membrane, placed between Teflon^®^ films and further dried in vacuum for 10 hours to remove residual solvent.

The weight fraction of TPU in the final composites was determined by weighing the dried nanocomposites. Details on how the composition of the SWCNT–TPU sheets was obtained as well as their characteristics (*e.g.*, density and volume fraction) are shown in Table S1. For sorption evaluation, the amount of TPU adsorbed per unit mass of CNTs (*C*_ads_) and its remaining concentrations in the solutions (*C*_eq_) at equilibrium were determined from:
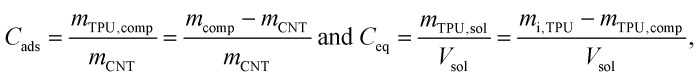
where *m*_CNT_ and *m*_i,TPU_ are the initial masses of CNTs and TPU, respectively, and *m*_comp_ corresponds to the mass of the dried nanocomposite. *m*_TPU,comp_, *m*_TPU,sol_ and *V*_sol_ correspond to the amount of TPU adsorbed by the CNTs, the amount of TPU remaining in solution, and the total volume of the solution, respectively (see Table S1).

SWCNT–BPs were obtained by vacuum filtration of the SWCNT suspension in the solvent/nonsolvent mixture. The suspensions were prepared by following the same steps for producing the nanocomposite sheets.

### 2.5. Characterization

Scanning electron microscopy (SEM) images were taken with a Hitachi High Technologies S-4800v microscope. Tensile testing was performed using an Instron 5900R load frame with a 500 N load cell and a displacement rate of 5 mm min^−1^. A minimum of five coupons (∼30 mm × 2 mm) of each material were tested. Electrical conductivity (*σ* = 1/*R*_s_*t*) was calculated from measurements of sheet resistance (*R*_s_), performed using a 4-point probe (Signatone SP4), and average thickness (*t*). X-ray photoelectron spectroscopy data were collected using a Kratos AXIS Ultra DLD spectrometer with a monochromated Al Kα beam (1486.6 eV) under high vacuum (5 × 10^−9^ torr). The data were analyzed using CasaXPS (version 2.3.17PR1.1), and the binding energy scale was calibrated by setting the primary C 1s peak to 284.5 eV for sp^2^ carbon in CNTs. X-ray diffraction (XRD) was performed using a Bruker D8 DISCOVER with Cu Kα_1_ radiation (*λ* = 1.5406 Å, 8.0478 keV). Data were collected in a 2-theta range of 5° to 90° with a scan speed of 0.02° per second. Water contact angle (WCA) was measured with an Attension optical tensiometer using the sessile drop method. A droplet of ultrapure MilliQ water was delivered onto the surface and 120 frames of images were recorded. Young–Laplace fitting method was used with manual baseline to acquire the WCA value at two different locations for each sample. Thermogravimetric analysis (TGA) was performed using a Netzsch STG 449 F1 instrument coupled with a Bruker Tensor 27 FTIR spectrometer. Approximately 10 mg of each sample was analyzed in argon atmosphere with a 10 °C min^−1^ heating rate.

## 3. Results and discussions

### 3.1. Mechanical properties and electrical conductivity of SWCNT–BP and nonwoven SWCNT–TPU composite sheets

**Fig. 1:**
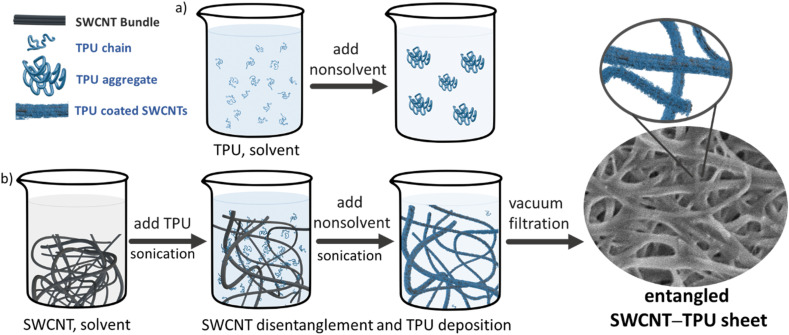
Schematics of the one-step vacuum filtration method. (a) TPU phase separation in the solvent/nonsolvent mixture. (b) Formation of coated SWCNTs by TPU deposition onto the SWCNTs surface and recovery of SWCNT–TPU nanocomposite sheets by vacuum filtration.

Fig. 1 illustrates the different stages of SWCNT disentanglement and TPU-coating for the fabrication of the nanocomposite sheets using the one-step filtration method. The solvent/nonsolvent volume ratio was empirically determined (see SI) and the SWCNT/TPU wt% ratio in the nanocomposite sheets is controlled by increasing the TPU concentration in solution while keeping the mass of SWCNTs (nominal specific surface area) constant. As previously shown, in the absence of CNTs, TPU chains phase separate upon combination with the nonsolvent (methanol) to form aggregates (Fig. 1a). In the presence of CNTs (Fig. 1b) both single polymer chains and TPU aggregates co-deposit on the surface of nanotubes, as the result of a different strength of CNTs–TPU, TPU–TPU and TPU–solvent interactions.^12,17^ Solution-based methods combine low viscosity, which facilitates polymer diffusion, with sonication to disentangle and debundle CNTs, thereby increasing surface area. The large specific surface area and one-dimensional structure of nanotubes provides abundant sites for TPU deposition and conformational arrangement.

**Fig. 2:**
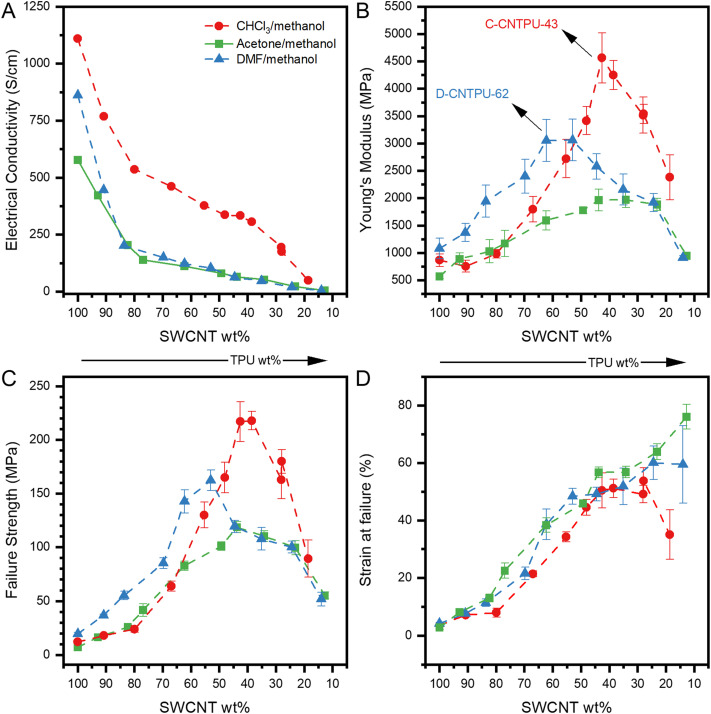
Electrical conductivity and mechanical properties of the SWCNT–TPU nanocomposite sheets and SWCNT–BPs (100 wt% SWCNTs) fabricated using different TPU solvents (acetone, DMF and CHCl_3_) and methanol as the nonsolvent (see Table S1 for compositional details). (A) Electrical conductivity. (B) Average Young's modulus. (C) Average failure strength and (D) average failure strain (lines to guide the eye).

Table S1 summarizes the characteristics of the SWCNT–BPs and nonwoven SWCNT–TPU sheets, with representative SEM images in Fig. S1. For practical applications, DMF is less desirable than acetone because of a higher boiling point; however, it has been widely used due to its high polarity and ability to interact with CNTs.^24^ Although CHCl_3_ is less commonly employed in nanocomposite fabrication, it can also be effective. Notably, CHCl_3_ and other halogenated species have been reported to dope SWCNTs, increasing carrier concentration and significantly enhancing the electrical conductivity of corresponding buckypapers.^25–27^ The electron withdrawal by adsorbed Cl and chemical intermediates can lead to p-type networks with high electrical conductivity. Fig. 2 summarizes the electrical conductivity and mechanical properties for SWCNT–TPU made from different solvent/nonsolvent mixtures. Electrical conductivity values were found to change significantly with time. Doping effects that influence the electrical properties of CNT assemblies due to exposure to ambient oxygen and water vapor have been reported.^28,29^ Fig. S2 presents conductivity variations measured over several months after fabrication, generally showing an increase with prolonged exposure to ambient conditions. For example, SWCNT buckypapers prepared in DMF/methanol exhibited an approximately 200% conductivity increase after 30 days. To account for such variations, Fig. 2A compares conductivities measured after 23–30 days of exposure.

Similar to MWCNT,^12^ replacing acetone with DMF increased SWCNT–BP conductivity (Fig. 2A). In contrast, for DMF-nanocomposites, electrical conductivity decreased drastically as TPU content increased, approaching the values obtained with acetone. DMF, being a more effective solvent for dispersing nanotubes than acetone, yields improvements in SWCNT–BP, which is attributed to smaller average bundle size and/or smaller average aggregate in solution. This results in a more dense packing in the recovered sheet, as reflected in Table S1 (density values of 0.32 g cm^−3^*vs.* 0.44 g cm^−3^ and SWCNT vol% of 18% *vs.* 23% for acetone and DMF, respectively). However, as TPU is incorporated, the differences in electrical conductivity between acetone-nanocomposites and DMF-nanocomposites become negligible. Although there are studies on the effect of different solvents on the dispersibility of SWCNTs, it is more difficult to predict the effect of both polymer and solvent on the state of SWCNT disentanglement and individualization, especially in a solvent/nonsolvent mixture.

Using CHCl_3_ in the solvent/nonsolvent mixture yielded buckypaper with conductivity of 1100 S cm^−1^, comparable to literature values,^26^ and produced nanocomposites with higher conductivity overall. Similar to the trends observed for DMF-nanocomposites, conductivity decreased markedly up to the 80 wt% SWCNT with TPU deposition, but the drop between 70 wt% and 40 wt% SWCNT was less pronounced (460 S cm^−1^ at 67 wt% and 300 S cm^−1^ at 38 wt%), suggesting that electrical transport within the nanocomposites was less affected by the increasing amount of insulating TPU in this range. At higher TPU contents, conductivity again fell more sharply. These results confirm more effective electric transport in nanocomposites fabricated using CHCl_3_ as the solvent.

Fig. 2B–D shows the Young's modulus, failure strength, and strain at failure of the fabricated buckypapers and nanocomposite sheets, with representative stress–strain curves in Fig. S3. Among the SWCNT–BPs, the highest failure strength, Young's modulus and SWCNT vol% (Table S1 and Fig. S4) were obtained for the SWCNT–BP fabricated using DMF, supporting the hypothesis that better nanotube disentanglement and smaller bundle size achieved with DMF lead to a more dense packing (higher SWCNT vol%) and greater inter-tube friction per unit volume.

Interestingly, CHCl_3_-nanocomposites also exhibited significantly higher mechanical properties. With the CHCl_3_/methanol mixture, mechanical properties increased as TPU was deposited onto the nanotubes, (*i.e.*, as the SWCNT wt% decreases), reaching a clear maximum at ∼40 wt% SWCNT (samples C-CNTPU-43 and C-CNTPU-38 in Table S1) before declining with further TPU incorporation. At 38 wt% SWCNT (16 vol%), the strength, stiffness and strain at failure reached 220 MPa, 4.2 GPa, and 50%, respectively, with a toughness of 77 MJ m^−3^ for (Fig. S5).

With acetone as the solvent, no clear optimal composition was observed; strength peaked at ∼120 MPa and remained similar across a wide SWCNT range (∼50 wt% to 25 wt%). Although a co-continuous TPU/SWCNT phase formed, which is evident by the significant improvement in mechanical properties as the TPU content increases, the characteristics of the nanocomposite network are not optimized. In contrast, DMF-nanocomposites showed a clearer optimal composition (∼60–50 wt% SWCNT; samples D-CNTPU-62 and D-CNTPU-53) and higher mechanical properties than acetone-nanocomposites. The use of a better SWCNT dispersant seems to lead to a better SWCNT disentanglement in solution and packing in the nanocomposite sheets.

Nevertheless, a better reinforcement effect is achieved for CHCl_3_-nanocomposites, reaching not only higher mechanical properties but also at lower SWCNT wt%. The highest strength (160 MPa) and Young's modulus (3 GPa) are reached at ∼50 wt% SWCNT (D-CNTPU-53, 19 vol%) with DMF, while with CHCl_3_ the maximum strength and Young's modulus are 50% and 35% higher, respectively, at ∼40 wt% SWCNT (C-CNTPU-43, 17 vol%). Unlike typical trade-offs, strain at break was maintained, resulting in a 45% increase in tensile toughness. Within the optimal composition, CHCl_3_-nanocomposites demonstrated 29-fold and 12-fold improvements in modulus and strength compared to pristine TPU, without significant toughness loss. This remarkable improvement in mechanical properties lead to the conclusion that the TPU–SWCNT interactions and conditions in the CHCl_3_/methanol solvent system must be improving the SWCNT–TPU interfacial attractions and/or the state of SWCNT disentanglement in solution.

The mechanical properties rank among the highest observed for CNT/polymer nanocomposites produced from CNT powders,^2,30^ and present a high performance, multifunctional nanocomposite through use of broadly available, powder-like SWCNTs of modest aspect ratio. Failure strength exceeded those of strong industrial plastics (20 MPa to 70 MPa) by at least one order of magnitude.^31^ Despite their high performance, the nanocomposite sheets remained low density, (0.4–0.9) g cm^−3^*vs.* TPU's 1.19 g cm^−3^, enhancing specific properties (Fig. S6). Failure strengths approached those of ultrahard cermets such as tantalum monocarbide (290 MPa) and silicon monocarbide (300 MPa), while maintaining far greater failure strain (∼50% *vs.* 0.2–0.6% in carbides).

### 3.2. Morphology and spectroscopic characterization of SWCNT–BP and nonwoven SWCNT–TPU composite sheets

**Fig. 3:**
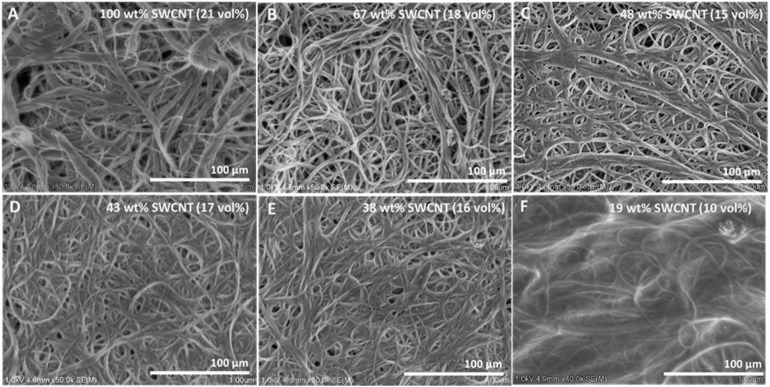
Representative SEM images of (A) SWCNT–BP and (B–F) SWCNT–TPU nanocomposite sheets of different compositions prepared with the CHCl_3_/methanol mixture (see Table S1 for compositional details).

Fig. 3 shows representative SEM images of SWCNT–BP and nonwoven SWCNT–TPU sheets of different compositions prepared with the CHCl_3_/methanol mixture (CHCl_3_-nanocomposites). Additional micrographs, including sheets fabricated with other solvent mixtures, are shown in Fig. S1. As previously reported, the characteristic disordered tangle of SWCNT bundles with a porous mesh structure and a wide distribution of bundle diameters is observed for the SWCNT–BP.^17^ The nanocomposites display a similar entangled, fiber-like morphology, with visible changes as TPU is deposited onto the nanotubes (Fig. 3B–F). In the nanocomposites, the observed bundle diameter reflects both the underlaying SWCNT bundle size and the thickness of the TPU coating along the nanotubes. Voids are present on the surface and tend to decrease with increasing TPU content, consistent with the density and void volume percentage trends in Table S1. Fig. 3D and E correspond to CHCl_3_-nanocomposites in the composition range that yielded the highest mechanical property improvements (Fig. 2; samples C-CNTPU-43 and C-CNTPU-38 in Table S1). In contrast, the sample with the highest TPU content examined (Fig. 3F) shows a markedly different morphology, along with substantial reductions in mechanical properties and electrical conductivity compared to the “optimal” composition (*e.g.*, Fig. 3D). Above the optimal TPU content, the fiber-like morphology becomes embedded in TPU, resulting in a pronounced reduction in visible voids. At this stage, “excess” free polymer coexists with TPU-coated SWCNTs.^12^

Accurate evaluation of the intertube and interbundle distances in SWCNT–TPU nanocomposite sheets from SEM images is challenging due to their entangled morphology and heterogeneity, including variation in TPU surface coverage, bundle size, and porosity. However, differences in the packing density of TPU-coated SWCNT in the nanocomposite sheets can be inferred by examining changes in SWCNT vol% as a function of SWCNT wt% (Fig. S4). A higher vol% at equivalent SWCNT wt% indicates a more dense packing. As shown in Fig. S4, particularly for nanocomposites fabricated with DMF and CHCl_3_, the SWCNT vol% remains relatively constant with increasing TPU content before decreasing more sharply. This decline occurs at SWCNT wt% below the optimal composition (*e.g.*, sample C-CNTPU-43 for CHCl_3_) and reflects an increase in the intertube/interbundle distance. This interpretation is consistent with the morphological changes seen in SEM (Fig. 3F) and with the drop in mechanical properties when TPU content exceeds the optimal composition. Dashed lines in Fig. S4 highlight that the decrease in SWCNT vol% occurs at higher SWCNT wt% in DMF-nanocomposites compared to CHCl_3_-nanocomposites, in line with the greater reinforcement observed in the latter.

Regarding changes in interfacial interactions, the surface modification of SWCNTs *via* species generated from chloroform sonolysis (*e.g.*, hydrogen chloride and chlorine gases)^32^ could favor stronger interactions with TPU. The mechanisms by which halide-containing molecules introduce p-doping in SWCNTs is not completely understood, with proposed mechanisms including the non-covalent bonding of halide molecules,^33^ covalent attachments of halogen adducts, and adsorption of chlorine anions.^34^ Kawasaki *et al.* described the concept of complex chemistry of carbon nanotubes,^35^ noting that anionic species from protonic acids such as HCl attach to positively charged CNTs to achieve charge compensation, with p-doped CNTs behaving as soft cations. Cl adatoms have been shown to increase a density of defect states near the Fermi level and cause cascading chemical adsorption of cationic gold, neutral gold clusters, and water.^36,37^ A modified Hückel calculation revealed excess charges to be localized in roughly 4 nm long charge puddles due to interactions with the adsorbed counterions.^38^

**Fig. 4:**
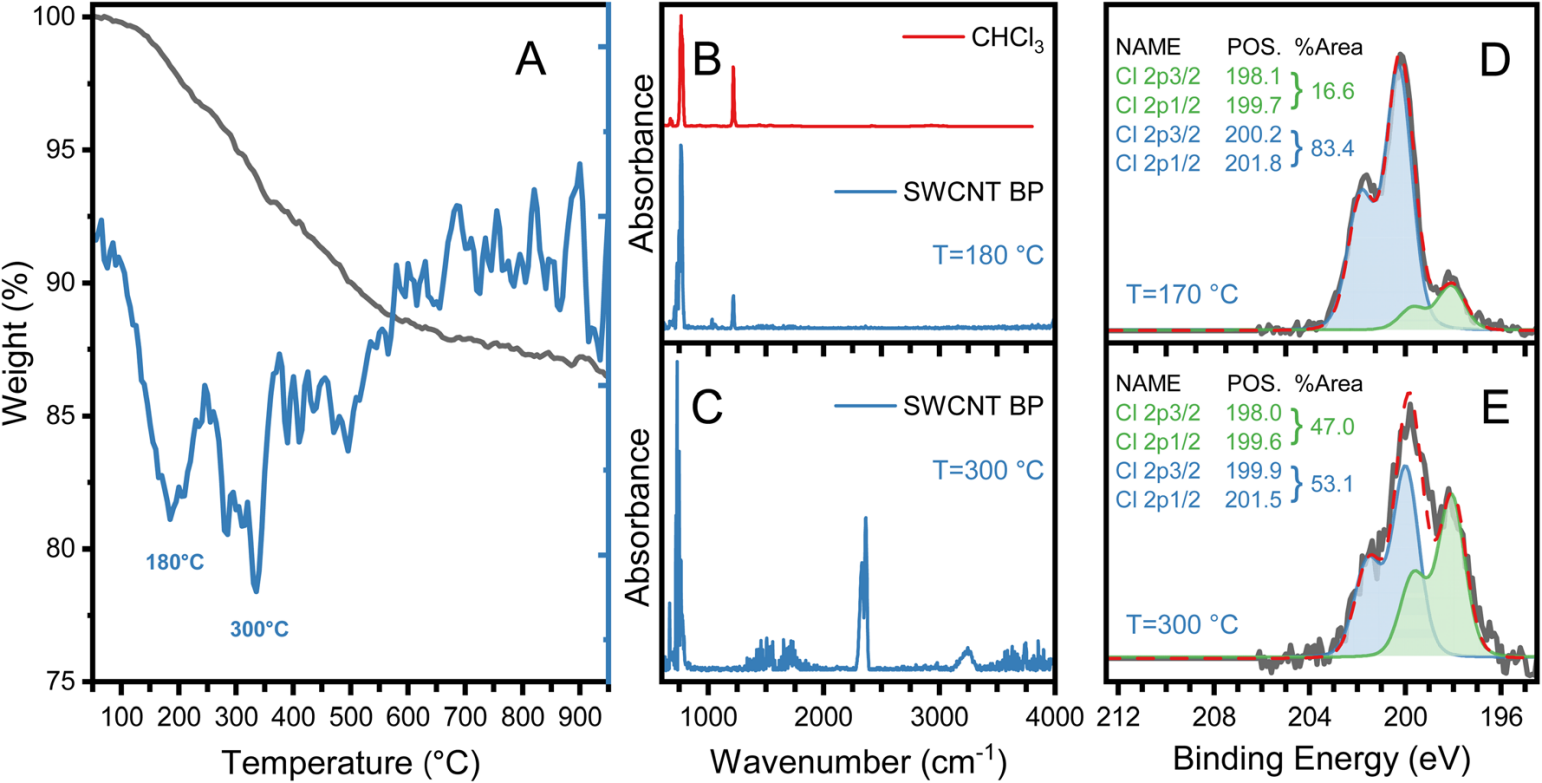
(A) TG (black) and DTG (blue) under desorption conditions for the SWCNT–BP prepared with CHCl_3_/methanol. (B) IR absorption spectrum of the species desorbed at 180 °C and the reference gas phase spectrum of CHCl_3_. (C) IR absorption spectra of the species desorbed at 300 °C. (D) and (E) XPS spectra (Cl 2p lines) for the SWCNT–BP after thermal treatments at 170 °C and 300 °C, respectively.

Surface modification of SWCNTs with chlorine atoms was confirmed in SWCNT–BPs fabricated with the CHCl_3_/methanol mixture using XPS. The results are shown in Fig. 4 (and Fig. S7), which also includes thermogravimetry and infrared absorption data (TGA-FTIR) obtained under argon atmosphere (desorption).

Thermogravimetric analysis of pristine SWCNT–BP obtained with the CHCl_3_/methanol mixture shows two mass-loss events (Fig. 4A): the first at 180 °C (∼3 wt%) attributed to physically adsorbed chloroform (Fig. 4B), and the second at ∼300 °C (∼4 wt%) corresponding to CO_2_ and water (Fig. 4C). The elevated desorption temperature of chloroform relative to its boiling point indicates strong halogenated solvent–nanotube interactions. No evidence of organic species generated by CHCl_3_ sonolysis was detected in the second event.

XPS analysis after drying at 170 °C (removing adsorbed CHCl_3_) revealed chlorine species at 1.4 at% and after a 300 °C treatment under vacuum, chlorine remained at 0.26 at% (Fig. S7). Fig. 4D and E show XPS spectra of Cl 2p lines for the samples treated at 170 °C and 300 °C, respectively. Two doublets (each consisting of the 2p_3/2_ and 2p_1/2_ pair with a spin–orbit splitting of 1.6 eV and 2 : 1 area ratio) are required to fit the Cl 2p spectrum in each sample. The binding energies of 2p_3/2_ components are approximately the same, 198.0 eV and 200.2 eV for the sample treated at 170 °C, and 198.0 eV and 199.9 eV for the sample treated at 300 °C. The two components are ascribed to inorganic chlorine (2p_3/2_ binding energy ∼198 eV), and organic chlorine (2p_3/2_ binding energy ∼200 eV).^39–41^ A binding energy of 198.0 eV has also been ascribed to atomic chlorine adsorbed on the surface.^42^ Notably, no changes in Raman D/G ratios were observed, indicating no new sp^3^ defects from C–Cl bond formation.

As shown in Fig. 4D and E, in addition to the reduction in the amount of chlorine (−80%), the relative intensity of the two doublets observed in the Cl 2p spectra changes with the thermal treatment. In the sample treated at 170 °C, the lower binding energy component at 198.0 eV corresponds to 17% of the total intensity of Cl 2p peak, whereas it comprises 47% of the intensity of Cl 2p peak for the sample treated at 300 °C, indicating that the chemical species corresponding to the higher binding energy component (an organic chlorine) decomposes or desorbs more with the 300 °C annealing than the lower binding energy inorganic chlorine component. Moonoosawmy *et al.*,^25^ assigned organic chlorine (∼200 eV) to adsorbed polymeric species generated during the sonolysis of halogenated solvent while ascribing the p-doping to iron chlorides. The desorption of Cl-modified organic species from the nanotubes could explain our results. However, in contrast with their observations and in agreement with Pelech *et al.*^40^ there were no changes in the nature of Fe species when comparing SWCNT–BPs fabricated with acetone and CHCl_3_ that would suggest formation of iron chlorides in the later (Fig. S7).

Electrical conductivity measurements (Table S2) revealed that removing physically adsorbed CHCl_3_ at 170 °C did not affect conductivity, indicating that residual adsorbed CHCl_3_ is not responsible for doping. After 300 °C annealing, conductivity decreased by ∼27% but remained higher than undoped SWCNT–BP (610 S cm^−1^*vs.* 280 S cm^−1^ in Table S2 for SWCNT–BP fabricated with CHCl_3_ and acetone, respectively). These results confirm a correlation between chlorine content (XPS) and conductivity, with chlorine atoms in different chemical environments strongly bound to SWCNTs and capable of doubling conductivity relative to undoped material. This surface modification could potentially enhance interfacial SWCNT–TPU interactions.

### 3.3. Adsorption behavior of TPU onto doped and undoped SWCNTs

The adsorption behavior by polymer chains dissolved and nanotubes dispersed in the same solvent has been scarcely studied and, like the case of adsorption on other solid surfaces, it has been mostly studied in ternary systems (dissolved polymer–nanotube–solvent).^43^ At equilibrium there is a defined distribution of the solute between the liquid and the solid phase, which can generally be expressed by an adsorption isotherm. For a system of CNTs (a sorbent) and a polymer solution, a graph of the solute (polymer) concentration in the solid phase, *C*_ads_ (mg g^−1^), can be plotted as a function of the solute concentration in the liquid phase, *C*_eq_ (mg L^−1^), at equilibrium.

**Fig. 5:**
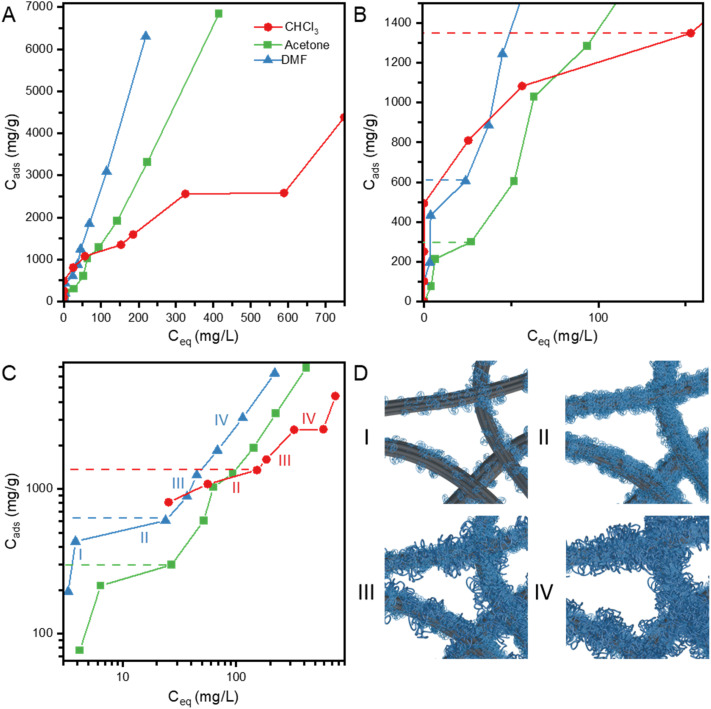
TPU concentration in the solid phase as a function of the TPU concentration in the liquid phase for three different solvent (DMF, acetone, and chloroform)/methanol mixtures: (A) full data range on a linear-linear scale, (B) low concentration range, (C) full range on a log–log scale. Roman numbers (I, II, III and IV) correspond to the different adsorption regions. (D) Cartoon representation of the different adsorption regions. Solid lines are shown to guide the eye and dashed lines to indicate TPU/SWCNT ratios (mg g^−1^) by the end of region II (samples in the optimal mechanical properties range in Fig. 2, D-CNTPU-62 and C-CNTPU-43 in Table S1).

The adsorption and co-deposition of TPU onto MWCNTs in a good TPU solvent (ternary system) as well as in a solvent/nonsolvent mixture was outlined in our previous study, demonstrating the advantages of the use of a solvent/nonsolvent combination for the fabrication of nanocomposites sheets.^12^ Unlike the former, a ternary system, the latter system is more complex since polymer solubility significantly differs between the solvent and the nonsolvent. Consequently, the overall polymer solubility in the mixed solvent/nonsolvent system decreases leading to some level of polymer aggregation. While the CNT–TPU–solvent–nonsolvent system is processed with high-speed dispersion, horn sonication, and bath sonication, dynamic equilibria are established at different stages of processing effectively leading to co-deposition onto nanotubes of both single polymer chains and aggregates as well as rearrangement of the adsorbed polymer. As the result, polymer deposition onto nanotubes can be expressed by an effective adsorption isotherm, which is a function of both the system composition and the processing conditions. Fig. 5A illustrates the adsorption behavior of TPU onto SWCNTs in the different solvent/nonsolvent mixtures, where *C*_ads_ (in mg of TPU per g of SWCNT) is plotted as a function of the TPU equilibrium concentration in the liquid phase (*C*_eq_), covering the wide TPU concentration range used for the nanocomposite fabrication.

The available SWCNT surface area may be different for different solvents since the nanotube dispersion state is unknown; different solvents can affect differently the degree of SWCNT aggregation and entanglement. DMF is considered a good dispersant for SWCNT, and the TPU adsorption observed here was more favorable with DMF as compared to acetone (*i.e.*, higher TPU adsorption at equivalent equilibrium concentrations). Nevertheless, at low TPU concentration (Fig. 5B, lower than ∼40 mg L^−1^), the adsorption was clearly more favorable with CHCl_3_. In fact up to the 1 : 0.5 ratio (SWCNT : TPU Table S1) all the TPU added was adsorbed resulting in liquid phase concentration being effectively equal to zero for the initial isotherm points. Using Giles *et al.*‘s classification of sorption isotherms,^44^ which is based on their initial slopes and curvatures, the adsorption in CHCl_3_/methanol presents an H (high affinity) behavior and convex shape. With DMF and acetone as the solvent, it was observed that the isotherms have a concave shape at low concentrations presenting an LS shape.^44^ The observed differences clearly demonstrate changes in the attractive forces of adsorption and a higher affinity of TPU for the nanotubes dispersed in the CHCl_3_/methanol mixture. As mentioned above, the doping of SWCNTs in CHCl_3_*via* sonochemically generated chlorine can modify the SWCNT surface. This surface modification must be responsible for enhanced attraction between the nanotubes and TPU leading to a more favorable adsorption. In semiconducting nanotubes (s-SWCNTs), shallow charge traps of a few nanometers in size, which have been described as “Coulomb defects” resulting from the interaction between charges on the s-SWNT and exohedral counterions, would create a localized electric field.^38^ Due to the electric field generated by the charge imbalance, Coulomb defects could create new electrostatic forces of attraction between the nanotubes and TPU. Simultaneously, this enhanced interaction could also lead to an improved SWCNT dispersion and disentanglement in solution, as sonication is applied at different stages, with a consequent increase in available surface area for TPU adsorption.

Fig. 5C shows the sorption data in a log–log scale, illustrating the step-wise behavior of the three isotherms. The adsorption of surfactants at the solid–liquid interface, which exhibits a step-wise behavior, has been explained using two models: the ‘two-step’ and ‘four-region’ adsorption models.^45,46^ Even though we cannot draw a direct analogy, we explore a model of different regions of TPU adsorption for the SWCNT/TPU system in a solvent/nonsolvent mixture. These regions are shown in Fig. 5C and D: a low surface coverage region (I), the first plateau region or layer completion (II), a multilayer growth region (III) and the second plateau (observed with CHCl_3_) or a lower rate of adsorption (observed for acetone and DMF) region (IV). In region I, the TPU (chains and/or small TPU aggregates) is expected to adsorb onto pristine SWCNTs mainly *via* van der Waal forces, which is the case with DMF and acetone used as solvents. With CHCl_3_, additional/higher binding energy adsorption sites are present probably due to the SWCNT surface modification with chlorine and the TPU uptake is higher as compared with the acetone and DMF systems. In region II the SWCNT surface attractive forces lead to saturation of all available adsorption sites and observation of the first plateau. It is evident that more TPU was required to complete region II (dashed lines in Fig. 5B and C) when CHCl_3_ was used as the solvent. The abrupt increase in adsorption denotes the onset of region III, where further increase in TPU concentration leads to the formation of multilayer structures and/or larger aggregates on the nanotube surface. The adsorption process in this region is probably significantly dominated by TPU–TPU interactions rather than SWCNT–TPU interactions, and TPU chains and/or aggregates that are already adsorbed *via* direct interaction with SWCNT sites act as anchors for aggregation of additional TPU chains (growth of the structures formed in region II). A region IV plateau in the TPU adsorption isotherm, observed only with CHCl_3_, indicates completion of higher surface coverage and formation of a fully developed multilayer. With acetone and DMF there is a decrease in the rate of adsorption in region IV, but adsorption increases monotonically. With CHCl_3_, TPU continuous adsorption is not observed in region IV due to more uniform first TPU layer coverage. The thermodynamic quality of the solvent (solvent/nonsolvent ratio) and TPU–TPU interactions are also expected to affect the onset of TPU aggregation and adsorption mechanisms.

### 3.4. The critical interphase

**Fig. 6:**
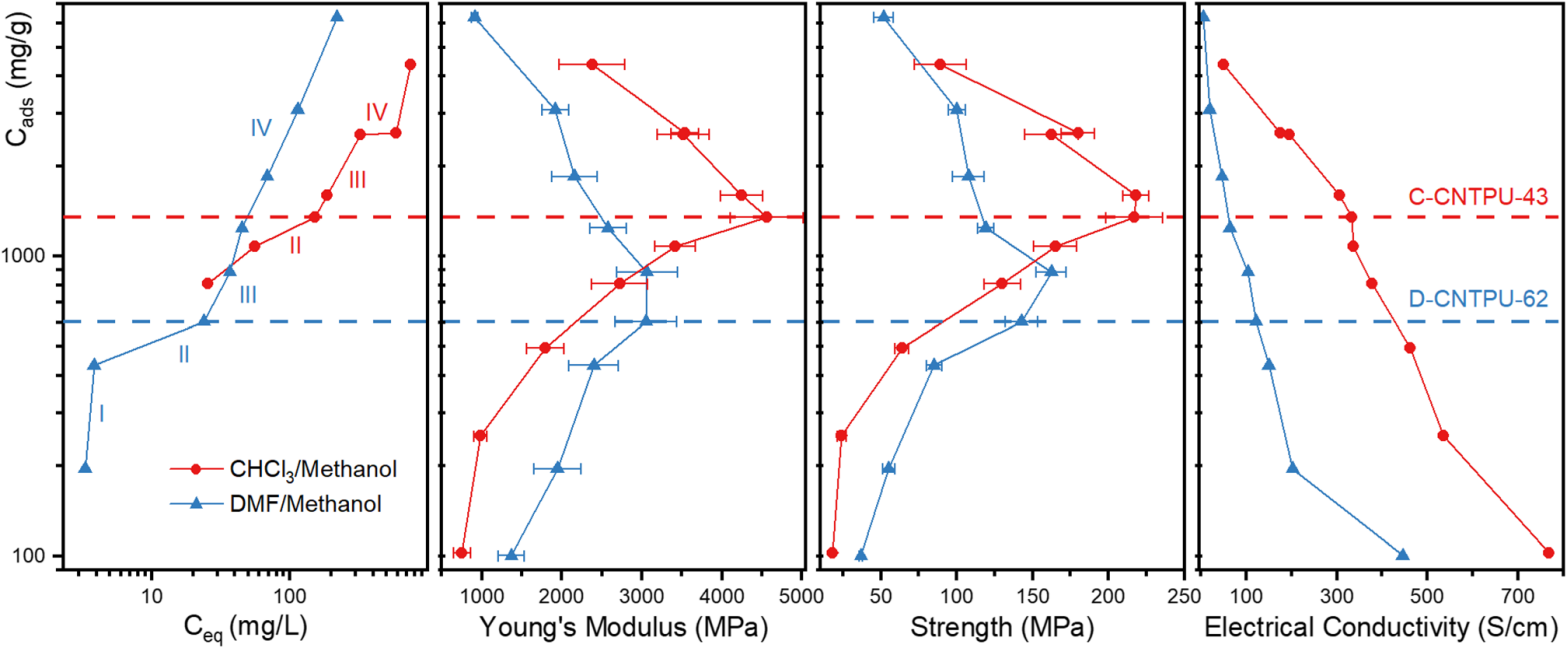
Correlation between the identified regions of adsorption with mechanical and electrical conductivity trends as a function of the amount of TPU adsorbed by SWCNT (mg g^−1^). Dashed lines to indicate TPU/SWCNT ratios by the end of region II and solid lines to guide the eyes.

Fig. 6 shows the correlation between the identified regions of adsorption with the mechanical and electrical conductivity trends as functions of the amount of TPU adsorbed by SWCNT (mg g^−1^). It can be clearly seen that compositions identified at the end of region II and beginning of region III (indicated by dashed lines in Fig. 6) correspond to samples with the highest improvements in mechanical properties (*e.g.*, D-CNTPU-62 and C-CNTPU-43 for DMF and CHCl_3_, respectively). These compositions correspond to saturation of SWCNT adsorption sites (end of region II) and onset of region III (multilayer region).

The analysis also demonstrates that with CHCl_3_, probably due to the SWCNT surface modification, new attractive forces led to stronger SWCNT–TPU interaction and higher TPU uptake up to Region II, and possibly an improved disentanglement of the SWCNTs in solution. The saturation of the available SWCNT surface area was achieved at 1350 mg g^−1^ and 600 mg g^−1^ for CHCl_3_ and DMF, respectively, which translates to a higher fraction of TPU strongly adsorbed at the interface for CHCl_3_-nanocomposites. Interestingly, for CHCl_3_-nanocomposites monotonic decrease of electrical conductivity with increasing TPU content locally slows down in the composition range identified as region II (65 wt% to 40 wt% SWCNT) showing an inflection, which could be explained by formation of a uniformly-packed layer of TPU on SWCNT surfaces preserving inter-tube average distance. The results indicate that formation of a uniformly packed SWCNT-coating layer due to the enhanced interaction between chlorine modified SWCNTs and TPU could lead to simultaneous enhancements in both mechanical properties and electrical conductivity. For both DMF and CHCl_3_, further TPU addition within the region III and beyond clearly led to a decrease in mechanical properties (*i.e.*, sample D-CNTPU-45 and C-CNTPU-27 in Table S1). This is consistent with significant decrease in the CNT vol% (Fig. S4 and Table S1) and increase in the inter-tube distance due to the formation of TPU multilayers. Increase in the intertube distance also leads to a decrease in electrical conductivity.

The analysis indicates that the SWCNT/TPU composition near the end of region II, where the higher mechanical properties were observed, correlates with the formation of a critical interphase characterized by a critical value of the interphase and the non-interphase ratio. This analysis of the adsorption behavior also supports the hypothesis that surface modification of SWCNTs with chlorine is an effective functionalization strategy to improve interfacial interactions and mechanical properties while also improving the electrical conductivity of TPU–SWCNT nanocomposites.

The effect of surface modification was also demonstrated by the fabrication of SWCNT–TPU sheets with the acetone/methanol mixture but using SWCNT that were first modified with chlorine (*via* sonication in CHCl_3_). In this case, as expected, nanocomposites (∼40 wt% SWCNT) recovered from the acetone/methanol mixture showed improvements with respect to equivalent sheets fabricated using unmodified nanotubes. With Cl-modified SWCNTs, the Young's modulus and failure strength increased by ∼30% while the electrical conductivity increased 3-fold.

### 3.5 Structure of TPU deposited onto SWCNTs

**Fig. 7:**
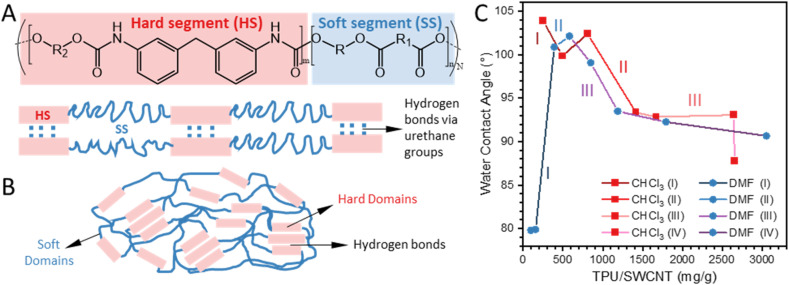
(A) General chemical structure of the TPU used in this study (MDI-based polyester-polyurethane) and schematic representation of hard and soft segments. (B) TPU phase separation to form soft and hard domains. (C) Average water contact angle of selected SWCNT–TPU nanocomposite sheets fabricated using different TPU solvents (DMF and CHCl_3_) with methanol as the nonsolvent.

The TPU used in this study is a segmented polyurethane, comprising alternating flexible and rigid chains in the polymer backbone. The hard segments (HS) derived from an aromatic diisocyanate, promote intermolecular hydrogen bonding (Fig. 7A), while the soft segments (SS) consist of a polyester. Due to chemical incompatibility between the two segment types, segmented polyurethanes exhibit microscale phase separation. Hard domains function as physical cross-links *via* reversible hydrogen bonding interactions through urethane groups. These hydrogen bonds, together with the hydrophilic/hydrophobic balance of TPU chains, are expected to play a significant role on the deposition and self-assembly of TPU chains and aggregates on nanotube surfaces, as well as on the interaction and entanglement between TPU-coated nanotubes.

Potential changes in the morphology or characteristics of deposited TPU and variations in TPU conformation or orientation are difficult to predict and characterize directly. However, static water contact angle (WCA) measurements could provide insights into the characteristics of the nanocomposite network (*i.e.*, TPU-coated SWCNTs) at different stages of TPU adsorption. Fig. 7C shows the WCA measured for selected nanocomposites with compositions corresponding to adsorption regions identified in Fig. 5, with additional data in Fig. S8, including WCA values for SWCNT–BPs. Fig. 7C reflects differences in the trends in WCA for nanocomposites with compositions identified as region I and II depending on the solvent used. With DMF the WCA for compositions in region I is ∼80°, while values of ∼100° and higher were observed in region I for nanocomposites fabricated with CHCl_3_. As the amount of TPU increases, and the available adsorption sites are saturated (region II), the WCA increases to ∼100° for DMF-nanocomposites but decreases for CHCl_3_-nanocomposites. Hence, for DMF-nanocomposites there is a change from mainly hydrophilic characteristics (WCA < 90°) in region I to hydrophobic (WCA > 90°) in region II. A different behavior is observed for CHCl_3_-nanocomposites as the TPU content increases.

The results suggest that Cl-modified nanotubes could be inducing changes on the conformation of TPU chains or aggregates deposited on the nanotubes, leading to less hydrophobic characteristics in CHCl_3_-nanocomposites by completion of region II as compared to DMF-nanocomposites. This could be explained by greater exposure of hard segment domains in TPU coatings for CHCl_3_-nanocomposites, which may also enhance mechanical properties through interphase crosslinking *via* hydrogen bonding interactions. Beyond region II, WCA gradually decreases for all samples as more TPU is deposited, indicating increased hydrophilicity, which correlates with the higher adhesion observed for sheets with the highest TPU content.^17,47^

**Fig. 8:**
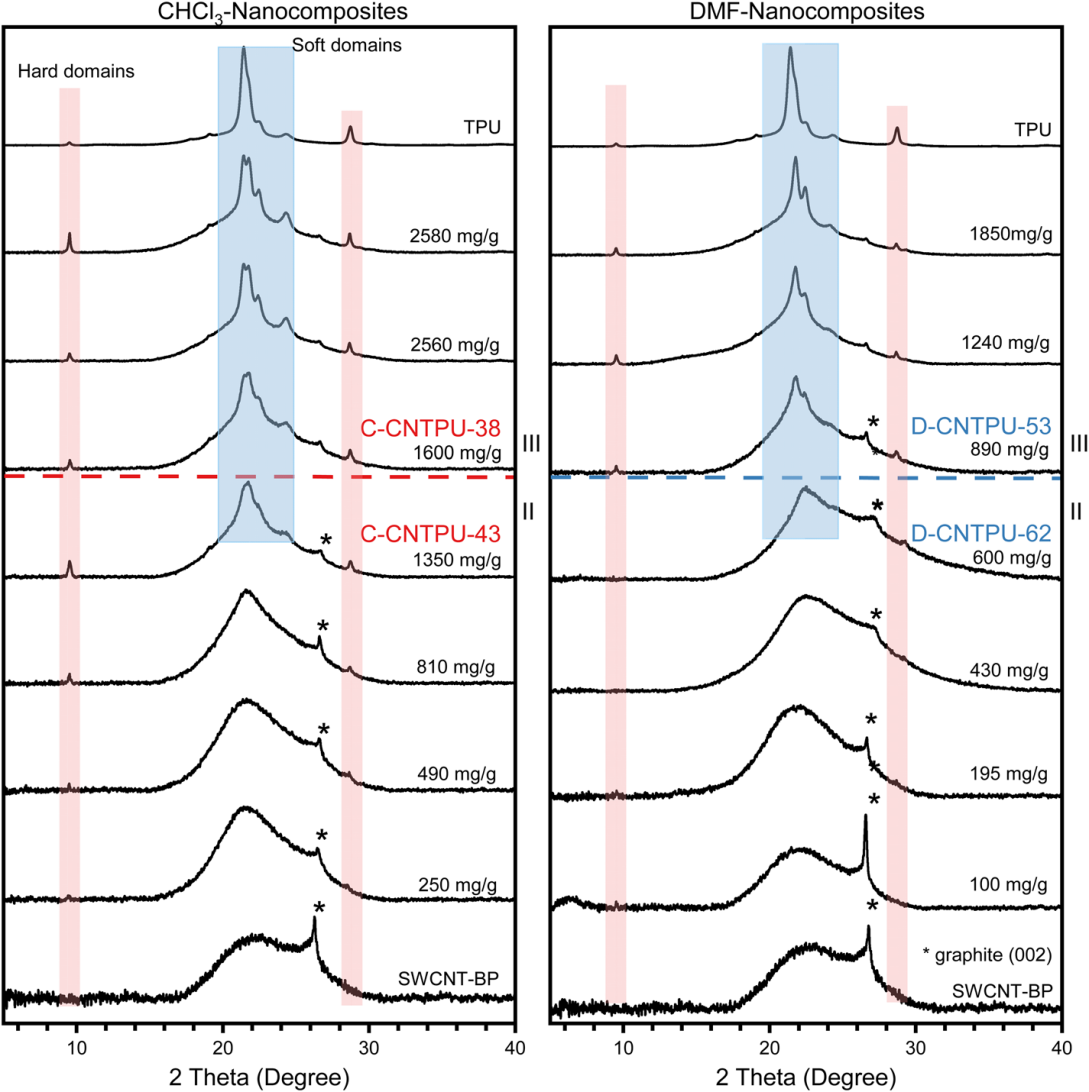
XRD patterns of TPU, SWCNT–BP, and SWCNT–TPU nanocomposite sheets fabricated using different TPU solvents (DMF and CHCl_3_) with methanol as the nonsolvent. Dashed lines to indicate TPU/SWCNT ratios (mg g^−1^) by the end of region II and transition to region III, corresponding to samples in the optimal mechanical properties range in Fig. 2 and 6. (*e.g.*, C-CNTPU-43 and D-CNTPU-62 in Table S1).

XRD was used to evaluate structural aspects of the polymer phase deposited onto the nanotubes at different compositions. The XRD patterns of TPU, SWCNT–BPs and nanocomposite sheets prepared with different solvent/nonsolvent mixtures, are presented in Fig. 8 and S9. All SWCNT–BPs exhibit a peak at 2*θ* ∼26.7°, consistent with the (002) diffraction peak of coarse graphite impurity and a halo at 22.7° that has been ascribed to a fine graphite-like impurity.^48,49^ TPU diffractograms feature sharp peaks at approximately 9.5°, 17.6°, 19.0°, 21.3°, 21.7°, 22.4°, 24.2°, 28.7°, along with a diffuse peak from about 15° to 25°, consistent with polyester-based polyurethane patterns reported previously.^50^ Peaks near 9.5° and 28.7° have been associated with the semi-crystalline hard domains, while the remaining peaks were associated with soft segment domains.^51^Fig. 8 compare CHCl_3_-nanocomposite DMF-nanocomposites as the amount of TPU increases (mg g^−1^). For clarity, the compositions corresponding to complete surface coverage (saturation of available adsorption sites) and transition from region II to region III (identified in Fig. 5 and 6) are also identified in Fig. 8. Below this transition, both CHCl_3_-nanocomposites and DMF-nanocomposites display a broad diffuse peak near 22° and no sharp peaks in that region, indicating the amorphous nature of adsorbed soft segments. In CHCl_3_-nanocomposites, however, HS-domain peaks are clearly visible for compositions below region II, suggesting aggregation of TPU chains into semi-crystalline HS domains (at least a few nanometers in size). As shown in Fig. S9, and as expected for semicrystalline polymers, there are some variations in repeated measurements. However, it appears that Cl-modified SWCNTs act as nucleation sites that promote HS crystallization compared to unmodified SWCNTs.

Interestingly, as TPU content increases beyond region II and TPU deposition transitions to region III, peaks corresponding to SS domains emerge and become increasingly sharper. This structural change is observed regardless of solvent choice, consistent with the proposed adsorption model and the identified compositions for saturation of adsorption sites. Comparing diffractograms for a DMF-nanocomposite with 1240 mg g^−1^ TPU (above complete coverage) and a CHCl_3_ nanocomposite with 1350 mg g^−1^ TPU (at completion of coverage) shows that SS peaks are not well defined in the latter, indicating that SS crystallization is only favored after adsorption site saturation. This aligns with the onset of multilayer growth in region III and the formation of thicker TPU coatings, which reduce SWCNT volume fraction and, consequently, mechanical properties (Fig. 6).

Across all nanocomposites, compositions corresponding to optimal mechanical properties—at the transition between regions II and III—still exhibit relatively disordered SS domains compared to neat TPU and higher TPU-content nanocomposite sheets. In CHCl_3_-nanocomposites, earlier HS crystallization suggests stronger HS–SWCNT interactions, enhancing interfacial load transfer and improving strength and stiffness without compromising SS flexibility for deformation and energy absorption. Furthermore, the inherent flexibility of SWCNT bundles promotes physical entanglement, while TPU chain entanglements and HS hydrogen bonding enables overlap and crosslinking. These combined effects strengthen long-range interactions within the SWCNT–TPU sheets, resulting in improved mechanical properties.

## 4 Conclusion

The electrical conductivity and mechanical properties of high-nanotube-content SWCNT–TPU nanocomposite sheets fabricated *via* the one-step filtration method were optimized by exploring different solvent/nonsolvent combinations for TPU. This versatile and practical method enables controlled TPU deposition onto nanotubes through solubility modulation, as demonstrated by effective adsorption isotherms. A stepwise adsorption model with multiple regions is proposed to describe TPU deposition onto nanotubes.

Mechanical properties exhibited a clear nonmonotonic trend with increasing TPU adsorption. The optimum in mechanical properties is attributed to the formation of a critical interphase. Analysis of the adsorption data identified the TPU/SWCNT ratio corresponding to saturation of SWCNT adsorption sites (end of region II in the effective adsorption isotherm), which coincides with the highest mechanical property improvements in the nanocomposite sheets (at ∼40 wt% SWCNT with CHCl_3_ and ∼60 wt% SWCNT with DMF). DMF, a more effective CNT dispersant, promoted deagglomeration and increased the available surface area for TPU deposition, resulting in greater TPU uptake and improved mechanical properties compared to acetone. Conversely, p-doping effects from CHCl_3_ significantly increased electrical conductivity and, through new attractive forces, increased TPU uptake beyond that achieved with DMF.

With CHCl_3_, stronger SWCNT–TPU interactions and a higher TPU/SWCNT wt% ratio at saturation promote more effective load transfer within the SWCNT–TPU network. At ∼38 wt% (16 vol%) SWCNTs, Young's modulus, failure strength, and strain reached 4.2 GPa, 220 MPa, and 50%, respectively, with tensile toughness of 77 MJ m^−3^. These values represent 50% and 35% increases in failure strength and modulus, respectively, compared to DMF-fabricated sheets at their optimal composition range. In the same composition range, electrical conductivity reached ∼300 S cm^−1^—three times higher than that obtained with DMF—highlighting the dominant role of SWCNT surface modification in CHCl_3_.

XRD results support the proposed adsorption model and indicate stronger interactions between TPU hard segments and SWCNTs, without compromising soft segment flexibility for deformation and energy absorption. Coated-SWCNT entanglement as well as TPU overlap and crosslinking likely promote long-range interactions, contributing to the outstanding mechanical performance.

The findings underscore the potential of the one-step filtration fabrication method and effective adsorption isotherm analysis for structural-functional integration optimization. This approach offers a robust method to control and study the effects of fabrication conditions, such as solvent type and SWCNT surface modification, on the properties of nanocomposites.

## Author contributions

Yadienka Martinez-Rubi: conceptualization, formal analysis, investigation, methodology, supervision, visualization, writing – original draft, writing – review & editing. Hao Li: conceptualization, investigation, methodology, validation, visualization, writing – review & editing. Kiran Mungroo: investigation, data curation, validation, writing – review & editing. Michael B. Jakubinek: conceptualization, formal analysis, methodology, writing – review & editing. Behnam Ashrafi: formal analysis, writing – review & editing. Zygmunt Jakubek: conceptualization, formal analysis, methodology, visualization, writing – original draft, writing – review & editing. Liliana Gaburici: data curation, investigation. Christopher Kingston: funding acquisition, supervision, visualization, writing – review & editing.

## Conflicts of interest

There are no conflicts of interest to declare.

## Supplementary Material

NA-008-D5NA00653H-s001

## Data Availability

The data supporting this article have been included in the manuscript and as part of the supplementary information (SI). Supplementary information: detailed composition and physical properties of the SWCNT–TPU sheets prepared. Specific mechanical properties of SWCNT–TPU sheets. Additional SEM images, XPS, XRD, water contact angle and electrical conductivity results. See DOI: https://doi.org/10.1039/d5na00653h.
