# Supplementary material for: Controlled adsorption of polyurethane onto chlorine-modified carbon nanotubes for enhanced mechanical and electrical properties of nanocomposites
Source: Nanoscale Adv. 2025 Oct 20;8(2):490–503. doi: 10.1039/d5na00653h (PMC12560951; doi:10.1039/d5na00653h)
Supplement: NA-008-D5NA00653H-s001 [file NA-008-D5NA00653H-s001.pdf]

Supplementary Information (SI) for Nanoscale Advances.
This journal is © The Royal Society of Chemistry 2025

Supporting Information

Controlled Adsorption of Polyurethane onto Chlorine-Modified Carbon Nanotubes for Enhanced Mechanical and Electrical Properties of Nanocomposites

Yadienka Martinez-Rubi[†*], *Hao Li*[†], *Kiran Mungroo*[†], *Michael B. Jakubinek*[†], *Behnam Ashrafi*[‡], *Zygmunt J. Jakubek*[§], *Liliana Gaburici*[†], *Christopher Kingston*[†]

[†] Quantum and Nanotechnologies Research Centre, National Research Council Canada, Ottawa, ON, Canada

[‡] Aerospace Research Centre, National Research Council Canada, Montreal, QC, Canada

[§] Metrology Research Centre, National Research Council Canada, Ottawa, ON, Canada

[*] Yadienka.Martinez-Rubi@nrc-cnrc.gc.ca

Preparation of SWCNT–BP and nonwoven SWCNT–TPU composite sheets

The solvent/nonsolvent volume ratio was empirically selected as to induce TPU phase separation (i.e., lowering the thermodynamic quality of the solvent to induce TPU chain aggregation and formation of a colloidal suspension). The total volume of the solvent mixture and the concentration of SWCNTs was constant for the different compositions (i.e, SWCNT:TPU wt. ratio) evaluated. The composition (i.e., SWCNT/TPU wt.% ratio) of the recovered nanocomposites was evaluated by weighing the nanocomposite sheets after drying at 75 °C. This way the amount of TPU adsorbed/coprecipitated onto the known initial mass of SWCNTs was determined. It is worth noting that TGA-FTIR analysis of SWCNT buckypapers (SWCNT–BP) and SWCNT–TPU sheets showed the presence of residual solvent after the drying step, especially for samples prepared with $CHCl_3$ and DMF. This was taken into account to correct the amount of TPU adsorbed by the SWCNTs (TPU w.t% in Table 1)

The external volume of the samples was evaluated from the SWCNT–TPU sheets. The thickness was measured with a Marathon Digital Electronic Micrometer having a resolution of 0.001 mm. The volume fraction of SWCNTs ($V_{f,CNT}$), TPU ($V_{f,TPU}$) and pores/voids ($V_{f,voids}$) in the samples were estimated using equations 1-3:

$$V_{f,CNT} = \frac{\rho_{Comp}}{\rho_{CNT}} W_{f,CNT}, \quad (1);$$

$$V_{f,TPU} = \frac{\rho_{Comp}}{\rho_{TPU}} W_{f,TPU}, \quad (2);$$

$$V_{f,voids} = 1 - V_{f,TPU} - V_{f,CNT}, \quad (3)$$

Where ρ_{Comp}, ρ_{CNT} and ρ_{TPU} are the densities of the nanocomposite sheet, SWCNTs, TPU, respectively. ρ_{Comp} was obtained by dividing the mass of the SWCNT–TPU sheets by their external volume, while a literature value of 1.8 g cm^{-3} value was used for ρ_{CNT}. $W_{f,CNT}$ and $W_{f,TPU}$ correspond to the weight fraction of SWCNTs and TPU in the SWCNT-TPU composite sheets, respectively.

Table S1. Characteristics of the SWCNT–BP (0 wt.% TPU) and nonwoven SWCNT–TPU nanocomposite sheets obtained using acetone (A-), DMF (D-) or $CHCl_3$ (C-) as the solvent and methanol as the nonsolvent. In the sample name the number corresponds to the SWCNT wt%. All samples were dried at 75 °C for 10 hours under vacuum before characterization

**Sample name**	**SWCNT/TPU wt. ratio in solution**	**Recovered SWCNT–TPU nanocomposite sheets**						
		SWCNT/TPU/X (wt% ratio)[a]	SWCNT/TPU (wt% ratio)[b]	TPU/SWCNT (mg/g)[b]	Density (g/cm^3)	Volume fraction (vol%)		
						SWCNT	TPU	void
**Acetone**								
A-SWCNT-100	1:0	94/0/6	100/0	0	0.32	18	0	82
A-CNTPU-93	1:0.1	92/7/1	93/7	77	0.37	19	2	79
A-CNTPU-82	1:0.25	81/17/2	82/18	215	0.36	16	6	78
A-CNTPU-77	1:0.5	76/23/1	77/23	300	0.44	18	9	73
A-CNTPU-62	1:1	61/38/1	62/38	604	0.53	18	17	65
A-CNTPU-49	1:1.5	49/50/1	49/51	1028	0.47	13	20	67
A-CNTPU-44	1:2	43/56/1	44/56	1285	0.60	14	28	57
A-CNTPU-34	1:3	34/65/1	34/66	1923	0.71	13	39	47
A-CNTPU-23	1:5	23/76/1	23/77	3315	0.89	11	58	31
A-CNTPU-13	1:10	13/87/0	13/87	6842	0.39	3	29	69
**DMF**								
D-SWCNT-100	1:0	92/0/8	100/0	0	0.44	23	4	74
D-CNTPU-91	1:0.1	84/8/8	91/9	100	0.50	23	7	70
D-CNTPU-84	1:0.25	79/16/5	84/16	195	0.47	21	8	71
D-CNTPU-70	1:0.5	66/29/5	70/30	434	0.57	21	16	63
D-CNTPU-62	1:1	59/36/5	62/38	606	0.66	22	23	56
D-CNTPU-53	1:1.5	50/45/5	53/47	886	0.67	19	28	53
D-CNTPU-45	1:2	44/54/2	45/55	1245	0.69	17	33	51
D-CNTPU-35	1:3	34/64/2	35/65	1849	0.82	16	45	39
D-CNTPU-24	1:5	24/74/2	24/76	3092	0.88	12	56	32
D-CNTPU-14	1:10	13/85/2	14/86	6302	0.86	6	63	31
**$CHCl_3$**								
C-SWCNT-100	1:0	85/0/15	100/0	0	0.44	21	6	74
C-CNTPU-91	1:0.1	82/8/10	91/9	102	0.40	18	6	75
C-CNTPU-80	1:0.25	72/18/10	80/20	251	0.43	17	10	73
C-CNTPU-67	1:0.5	63/31/6	67/33	493	0.51	18	16	66
C-CNTPU-55	1:1	53/43/4	55/45	809	0.56	17	22	61
C-CNTPU-48	1:1.5	46/50/4	48/52	1082	0.59	15	26	58
C-CNTPU-43	1:2.5	41/56/3	43/57	1348	0.73	17	36	48
C-CNTPU-38	1:3	37/60/3	38/62	1596	0.76	16	40	45
C-CNTPU-28	1:5	27/70/3	28/72	2563	0.78	12	48	40
C-CNTPU-28	1:7	27/71/2	28/72	2582	0.83	13	50	37
C-CNTPU-19	1:10	18/80/2	19/81	4381	1.02	10	70	20

[a] X corresponds to the wt.% of non-TPU adsorbed species, as determined by TGA-FTIR (i.e., desorbed species at temperatures below the TPU decomposition temperature, for example, residual solvent). [b] Corresponding to “dry solids” (corrected for non-TPU adsorbed species)

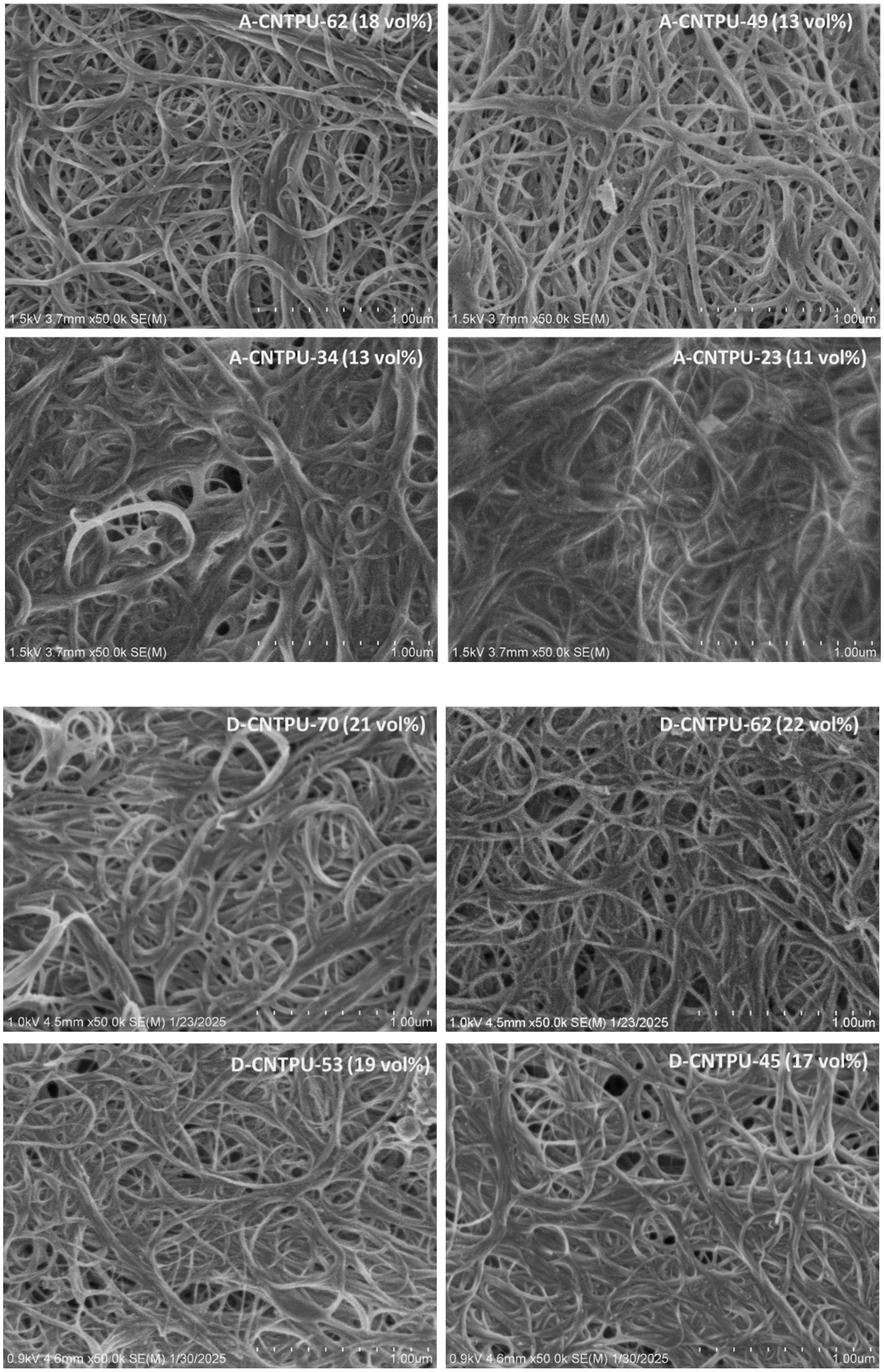

Figure S1. Representative SEM images of acetone-nanocomposites and DMF-nanocomposites (See Table S1 for sample details)

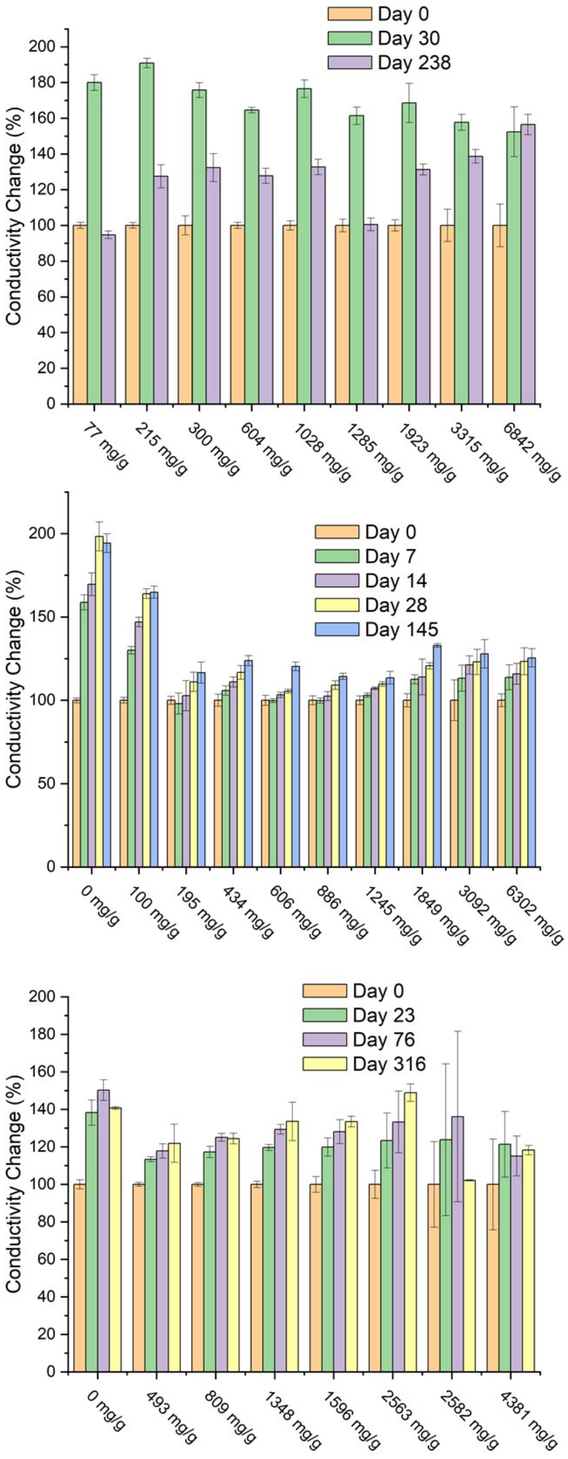

Figure S2. Changes in electrical conductivity with time for SWCNT-BPs and SWCNT-TPU nanocomposites fabricated using different TPU solvents (acetone, DMF or CHCl3) and methanol as the nonsolvent.

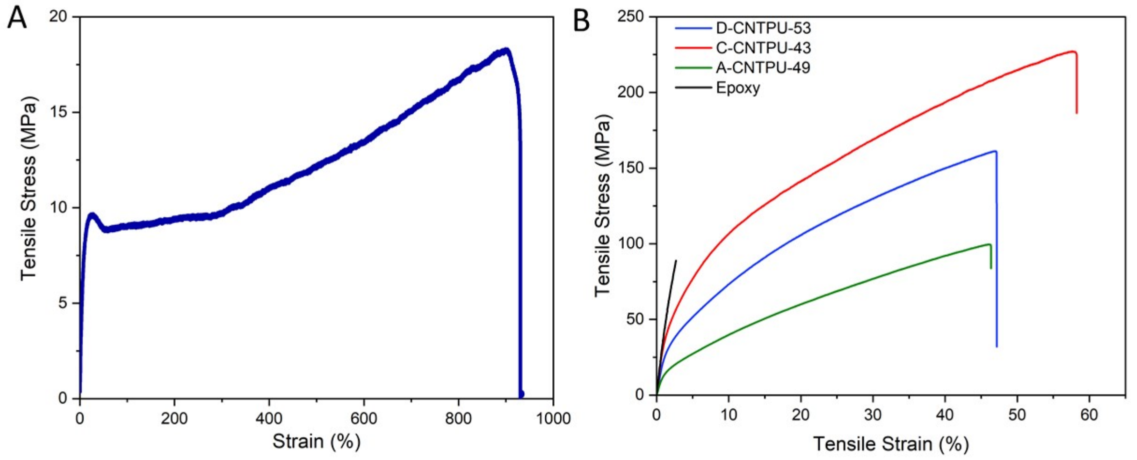

Figure S3. Representative stress–strain curves for A) Pristine TPU B) nonwoven SWCNT–TPU composite fabricated with different solvent/nonsolvent mixtures (Table S1) and an epoxy matrix.

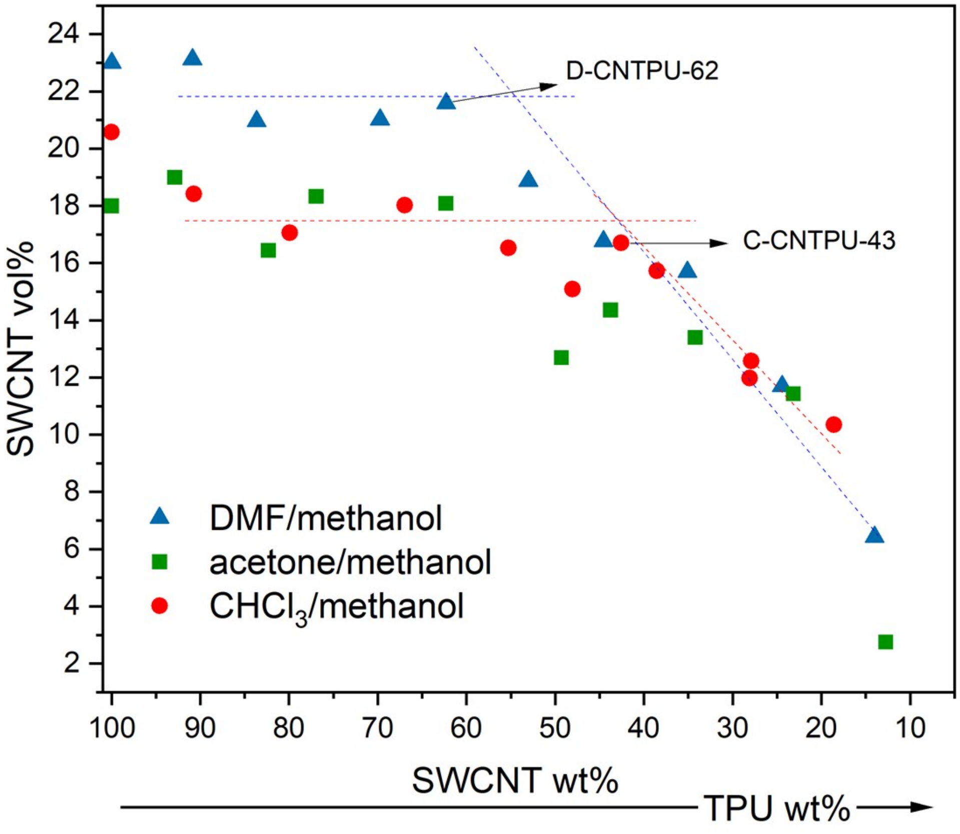

Figure S4. Changes in the SWCNT vol% as a function of the SWCNT wt.% for SWCNT-BPs and nanocomposites fabricated with different solvent/nonsolvent mixtures.

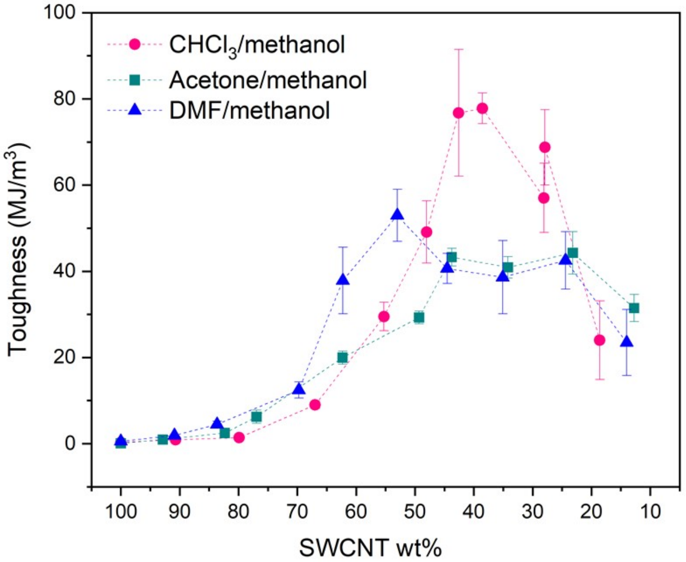

Figure S5. Tensile toughness of the SWCNT–TPU composite sheets and SWCNT–BPs fabricated using different TPU solvents (acetone, DMF and CHCl3) and methanol as the nonsolvent (see Table S1 for compositional details).

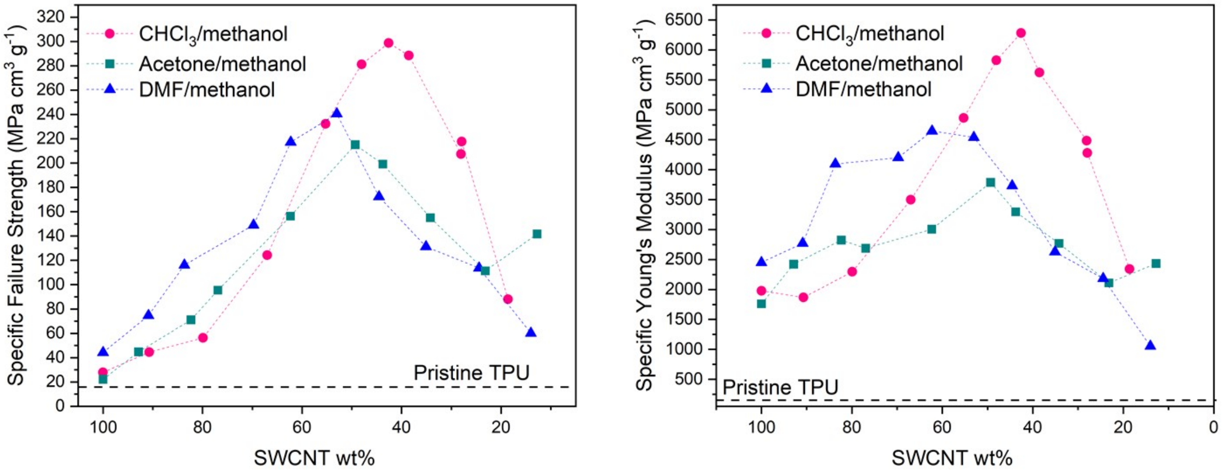

Figure S6. Specific mechanical properties of the SWCNT–TPU nanocomposite sheets and SWCNT–BPs fabricated using different TPU solvents (acetone, DMF and $CHCl_3$) and methanol as the nonsolvent (see Table S1 for compositional details). (A) Average failure strength (C) Average Young's modulus. (Lines to guide the eye).

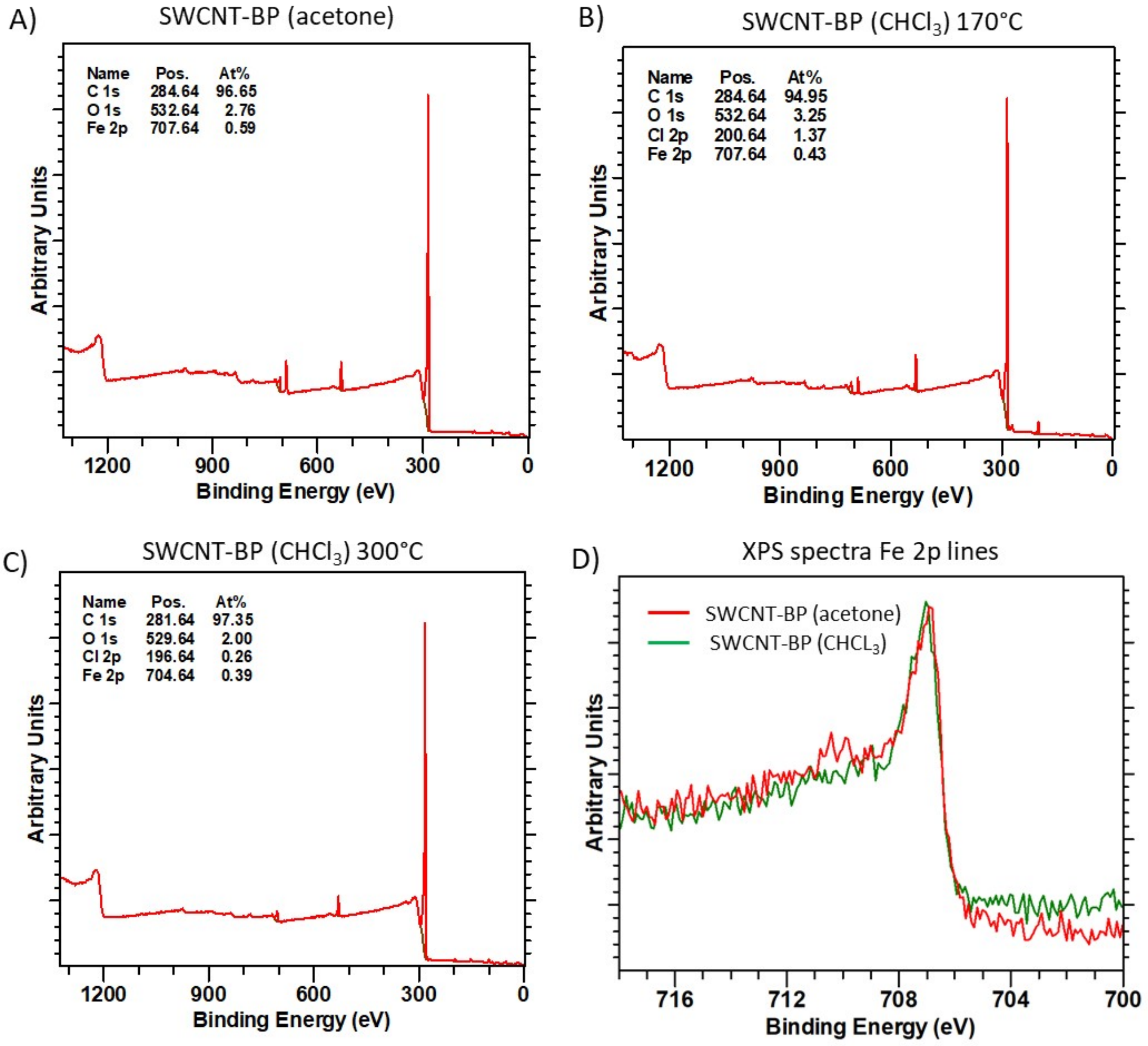

Figure S7. XPS spectra of SWCNT–BPs fabricated with different solvent/nonsolvent mixtures. A) acetone/methanol. B) $CHCl_3$/methanol treated at 170 °C. C) $CHCl_3$/methanol treated at 300 °C. D) High-resolution spectra of Fe 2p lines.

Table S2. Electrical conductivity of SWCNT-BP fabricated with $CHCl_3$/methanol and acetone/methanol before and after different thermal treatments (170 °C for 3h in air and 300 °C for 2h in vacuum). The electrical conductivity was measured months after storing samples in ambient conditions, before and immediately after the 170 °C treatment, and subsequently after a week in ambient conditions.

	Electrical conductivity (S/cm)			
Sample	Ambient Conditions[1]	Immediately After 170 °C	Ambient Conditions[2]	Immediately After 300 °C
SWCNT-BP (acetone)	580	260	580	281
SWCNT-BP ($CHCl_3$)	1210	825	1220	605

[1]months after fabrication
[2]one week after thermal treatment

The electrical conductivity of SWCNT-BP ($CHCl_3$) and SWCNT-BP (acetone) samples decrease after the 170 °C treatment. However, the values are recovered after a week of storing the samples in ambient conditions. This demonstrates a level of doping in ambient conditions for both samples. The decrease in electrical conductivity after the 170 °C treatment is probably due to removal of oxygen/moisture doping, which is recovered after reconditioning the samples in ambient conditions. The behaviour observed for the SWCNT-BP ($CHCl_3$) sample suggests a combination of oxygen and chlorine p-doping upon re-exposure to ambient atmosphere.

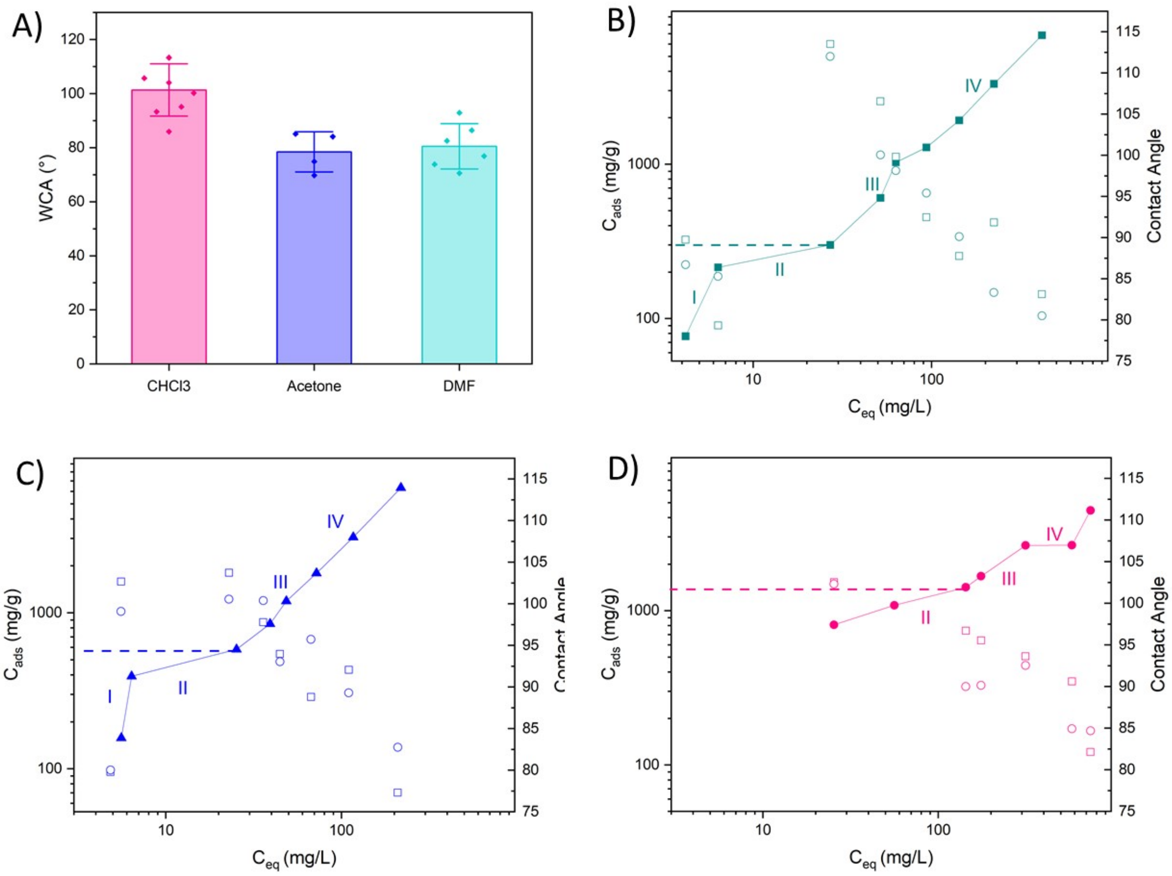

Figure S8. A) Water contact angle of pristine buckypapers fabricated with different methanol/solvent (i.e., acetone, DMF or $CHCl_3$) mixtures. Combined water contact angle and adsorption data of TPU onto SWCNTs for nanocomposites fabricated with different methanol/solvent mixture: A) Acetone, B) DMF and C) $CHCl_3$. Roman numbers (I, II, III and IV) correspond to the different identified adsorption regions. Continuous lines to guide the eye and dashed lines to indicate samples corresponding to completion of region II. Two data points for water contact angle (circles and squares) were collected from different areas in the sample and average values are reported in Figure 6.

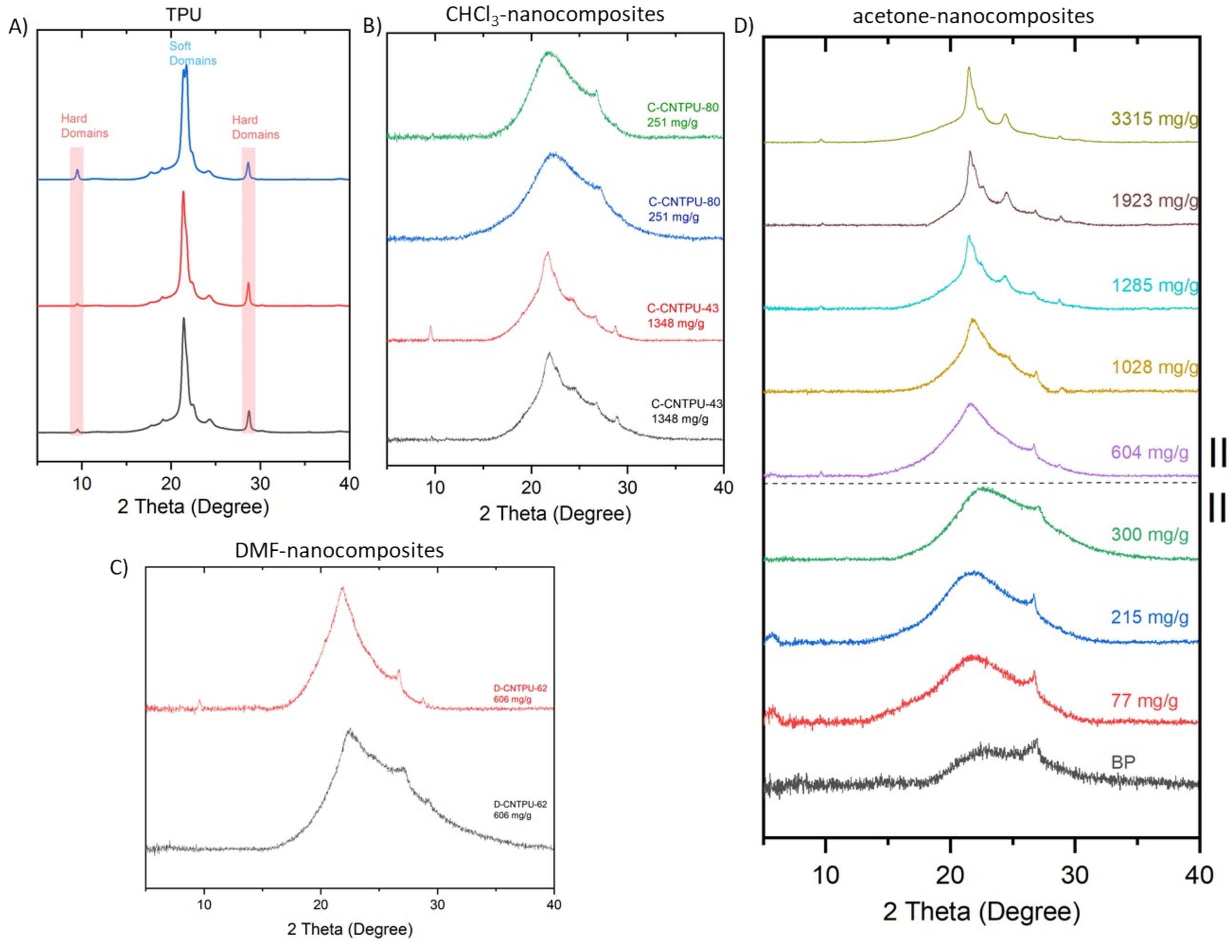

Figure S9. XRD diffraction patterns of A) TPU, B) repeat measurements on selected $CHCl_3$-nanocomposites, C) repeat measurements on selected DMF-nanocomposites D) acetone-nanocomposite sheets, dashed lines to indicate TPU/SWCNT ratios (mg/g) by the end of region II and transition to region III.
